# Supplementary material for: HIVprotI: an integrated web based platform for prediction and design of HIV proteins inhibitors
Source: J Cheminform. 2018 Mar 9;10:12. doi: 10.1186/s13321-018-0266-y (PMC5845081; doi:10.1186/s13321-018-0266-y)
Supplement: Supplementary file 2 — Additional file 2. Source code of HIVProtI web server. [file 13321_2018_266_MOESM2_ESM.zip › HIVprotI_Source-code/hivprotI/weka-3-6-6/doc/weka/classifiers/trees/ft/FTLeavesNode.html]

FTLeavesNode


---


|  |  |  |
| --- | --- | --- |
| **Overview** \| **Package** \| **Class** \| **Tree** \| **Deprecated** \| **Index** \| **Help** \| **Weka's home** | |  |
| **PREV CLASS**   **NEXT CLASS** | **FRAMES**    **NO FRAMES**     **All Classes** |  |
| SUMMARY: NESTED \| FIELD \| CONSTR \| METHOD | DETAIL: FIELD \| CONSTR \| METHOD |  |


---


## weka.classifiers.trees.ft Class FTLeavesNode

```
java.lang.Object
  weka.classifiers.Classifier
      weka.classifiers.trees.lmt.LogisticBase
          weka.classifiers.trees.ft.FTtree
              weka.classifiers.trees.ft.FTLeavesNode
```

**All Implemented Interfaces:**: java.io.Serializable, java.lang.Cloneable, CapabilitiesHandler, OptionHandler, RevisionHandler, WeightedInstancesHandler

---

``` public class FTLeavesNode extends FTtree ```

Class for Functional Leaves tree version.

**Version:**
:   $Revision: 1.4 $

**Author:**
:   Jo\~{a}o Gama, Carlos Ferreira

**See Also:**: Serialized Form

---

**Constructor Summary**

`FTLeavesNode(boolean errorOnProbabilities,
int numBoostingIterations,
int minNumInstances,
double weightTrimBeta,
boolean useAIC)`
  
          Constructor for Functional Leaves tree node.


| **Method Summary** | |
| --- | --- |
| `void` | `buildClassifier(Instances data)`             Method for building a Functional Leaves tree (only called for the root node). |
| `void` | `buildTree(Instances data, SimpleLinearRegression[][] higherRegressions, double totalInstanceWeight, double higherNumParameters)`             Method for building the tree structure. |
| `double[]` | `distributionForInstance(Instance instance)`             Returns the class probabilities for an instance given by the Functional Leaves tree. |
| `java.lang.String` | `getRevision()`             Returns the revision string. |
| `double` | `prune()`             Prunes a tree using C4.5 pruning procedure. |

**Methods inherited from class weka.classifiers.trees.ft.FTtree**

`assignIDs, assignLeafModelNumbers, cleanup, getConstError, getModelParameters, getNodes, getNodes, getNumInnerNodes, getNumLeaves, graph, hasModels, modelDistributionForInstance, modelsToString, numLeaves, numNodes, toString`

**Methods inherited from class weka.classifiers.trees.lmt.LogisticBase**

`getMaxIterations, getNumRegressions, getUseAIC, getUsedAttributes, getWeightTrimBeta, percentAttributesUsed, setHeuristicStop, setMaxIterations, setUseAIC, setWeightTrimBeta`

**Methods inherited from class weka.classifiers.Classifier**

`classifyInstance, debugTipText, forName, getCapabilities, getDebug, getOptions, listOptions, makeCopies, makeCopy, setDebug, setOptions`

**Methods inherited from class java.lang.Object**

`equals, getClass, hashCode, notify, notifyAll, wait, wait, wait`

**Constructor Detail**

### FTLeavesNode

```
public FTLeavesNode(boolean errorOnProbabilities,
                    int numBoostingIterations,
                    int minNumInstances,
                    double weightTrimBeta,
                    boolean useAIC)
```

:   Constructor for Functional Leaves tree node.

    **Parameters:**: `errorOnProbabilities` - Use error on probabilities for stopping criterion of LogitBoost?: `numBoostingIterations` - sets the numBoostingIterations parameter: `minNumInstances` - minimum number of instances at which a node is considered for splitting


**Method Detail**

### buildClassifier

```
public void buildClassifier(Instances data)
                     throws java.lang.Exception
```

:   Method for building a Functional Leaves tree (only called for the root node).
    Grows an initial Functional Tree.

    :   **Specified by:**: `buildClassifier` in class `FTtree`
    :   **Parameters:**: `data` - the data to train with **Throws:**: `java.lang.Exception` - if something goes wrong

---


### buildTree

```
public void buildTree(Instances data,
                      SimpleLinearRegression[][] higherRegressions,
                      double totalInstanceWeight,
                      double higherNumParameters)
               throws java.lang.Exception
```

:   Method for building the tree structure.
    Builds a logistic model, splits the node and recursively builds tree for child nodes.

    :   **Specified by:**: `buildTree` in class `FTtree`
    :   **Parameters:**: `data` - the training data passed on to this node: `higherRegressions` - An array of regression functions produced by LogitBoost at higher levels in the tree. They represent a logistic regression model that is refined locally at this node.: `totalInstanceWeight` - the total number of training examples: `higherNumParameters` - effective number of parameters in the logistic regression model built in parent nodes **Throws:**: `java.lang.Exception` - if something goes wrong

---


### prune

```
public double prune()
             throws java.lang.Exception
```

:   Prunes a tree using C4.5 pruning procedure.

    :   **Specified by:**: `prune` in class `FTtree`
    :   **Throws:**: `java.lang.Exception` - if something goes wrong

---


### distributionForInstance

```
public double[] distributionForInstance(Instance instance)
                                 throws java.lang.Exception
```

:   Returns the class probabilities for an instance given by the Functional Leaves tree.

    :   **Specified by:**: `distributionForInstance` in class `FTtree`
    :   **Parameters:**: `instance` - the instance **Returns:**: the array of probabilities **Throws:**: `java.lang.Exception` - if distribution can't be computed successfully

---


### getRevision

```
public java.lang.String getRevision()
```

:   Returns the revision string.

    :   **Specified by:**: `getRevision` in interface `RevisionHandler` **Overrides:**: `getRevision` in class `FTtree`
    :   **Returns:**: the revision


---


|  |  |  |
| --- | --- | --- |
| **Overview** \| **Package** \| **Class** \| **Tree** \| **Deprecated** \| **Index** \| **Help** \| **Weka's home** | |  |
| **PREV CLASS**   **NEXT CLASS** | **FRAMES**    **NO FRAMES**     **All Classes** |  |
| SUMMARY: NESTED \| FIELD \| CONSTR \| METHOD | DETAIL: FIELD \| CONSTR \| METHOD |  |


---
